# Supplementary material for: Chromothripsis during telomere crisis is independent of NHEJ, and consistent with a replicative origin
Source: Genome Res. 2019 May;29(5):737–49. doi: 10.1101/gr.240705.118 (PMC6499312; doi:10.1101/gr.240705.118)
Supplement: Supplemental Material [file supp_gr.240705.118_Supplemental_file_1.zip › contigs/annotated_contigs/DB46/contig.2.DB46_length_740_mean_cov_8.17027027027.docx]

**DB46_length_740_mean_cov_8.17027027027**

```
GCGTTTGCATCCTCATAGCTTAGCTCCCACTTCTAAGTGAGAACATATGGTATTTGGTTTTCCATTCCTGAGTTACTTCACTTAGACTA
 >chr18:57649764-57650087 + E=2e-167
ATTACCTCCATTTCCAACCAAGTTACTGCAAAAGACTTTTTTTTTTTATTTGATTAGTATTCCATGGTTTATATATGCCACATTTTCTT

TACCCACTCATCAGATGACGTGCACTTAGATTGGCTCCATATCTTTGCAGTTGTGACTTGTAGACTGGTGCTGCAATAAACATATGTGT

ACAGGTGTCTTTTTAATGTAGTGACTTCTTTTCCTTTGGGTAGAGGCCAGCGATAT|ACCTTCTGAGCATAAAAGCAACATGCTGCTGC
                                                          >chr18:57653044-57653461 - E=5
TTCTCTTCCGCCTTCCAAATCTCGTACATCAGTGTCTCTTCTGGACCACACTAACTATGCAGGAAACTCTGCAGGGAAGGGAATTCAGG
e-227
GAACTATCATTCCAGCTTAGCTTCATTGACTCAATACAAATCTACTACATCAAGTTTGATGATTCGGTTTTGTTTTACCTGCGGGTGAA

TCTACTCATTACTGCAAGGGAGTACCAGGAGTCTCACACAGGCAAGTGTAGCACACTCAAGACCTTTCCTTGGCCAGACGCGGTGGCTC

ATGCCTGTAATCCCAGCCCTTTGGGAGGGTGAGGCGGGCAGATCACAAGGTCGGGAGTTTGAGACAAGTCTGGCCAACATGGTGAAACT

CCATCTCTACTAAAAATACAAAAATTAGC
```
